# Spatiotemporal Activity Patterns of Sympatric Rodents and Their Predators in a Temperate Desert-Steppe Ecosystem

**DOI:** 10.3390/ani15152290

**Published:** 2025-08-05

**Authors:** Caibo Wei, Yijie Ma, Yuquan Fan, Xiaoliang Zhi, Limin Hua

**Affiliations:** 1 Key Laboratory of Grassland Ecosystem of the Ministry of Education, Engineering and Technology Research Center for Alpine Rodent Pest Control of National Forestry and Grassland Administration, College of Grassland Science, Gansu Agricultural University, Lanzhou 730070, China; weicb@st.gsau.edu.cn (C.W.); petrichor.yuquan@gmail.com (Y.F.); maccazhi@126.com (X.Z.); 2 Gansu Forestry and Grassland Administration, Lanzhou 730070, China; mayj_gsau@163.com

**Keywords:** desert-steppe ecosystem, rodent activity patterns, Infrared camera traps, predator–prey interaction

## Abstract

Understanding how prey and predator species partition activity patterns across time and space is essential for elucidating behavioral adaptation and ecological coexistence. In this study, we examined the diel and seasonal activity rhythms of two sympatric rodent species—*Rhombomys opimus* (Great gerbil) and *Meriones meridianus* (Midday gerbil)—and their primary predators, *Otocolobus manul* (Pallas’s cat) and *Vulpes vulpes* (Red fox), in a desert-steppe ecosystem on the northern slopes of the Qilian Mountains, China. Using over 8000 camera trap days and kernel density estimation, we quantified their activity intensity and spatiotemporal overlap. The two rodent species showed clear temporal niche differentiation but differed in their synchrony with predators. *R. opimus* exhibited a unimodal diurnal rhythm with spring activity peaks, while *M. meridianus* showed stable nocturnal activity with a distinct autumn peak. Notably, *O. manul* adjusted its activity pattern to partially align with that of *R. opimus*, whereas *V. vulpes* maintained a crepuscular–nocturnal rhythm overlapping more closely with that of *M. meridianus*. Despite distinct temporal rhythms, both rodent species shared high spatial overlap with their predators (overlap index OI = 0.64–0.83). These findings suggest that temporal partitioning may reduce predation risk for *R. opimus*, while *M. meridianus* co-occurs more extensively with its predators. Our results highlight the ecological role of native carnivores in rodent population dynamics and support their potential use in biodiversity-friendly rodent management strategies under arid grassland conditions.

## Simple Summary

Understanding when and where animals are active helps reveal how they coexist and interact. This study used infrared camera traps to investigate the daily and seasonal activity patterns of two coexisting desert rodents—Great gerbil (*Rhombomys opimus*) and Midday gerbil (*Meriones meridianus*)—and their main predators, Pallas’s cat (*Otocolobus manul*) and Red fox (*Vulpes vulpes*), in a temperate desert-steppe of northern China. The two rodents showed distinct activity rhythms, which likely reduce competition. Despite this, both overlapped substantially in space and time with their predators. Notably, *O. manul* showed flexible activity aligned with that of *R. opimus*. These findings improve our understanding of rodent–predator dynamics and suggest that enhancing natural predators could be a biodiversity-friendly strategy for rodent control in arid grasslands.

## 1. Introduction

Animal activity patterns lie at the heart of behavioral ecology, reflecting adaptive strategies shaped by both genetic mechanisms and environmental pressures. Generally, activity patterns comprise two primary components: activity rhythms and activity intensity [1,2,3,4]. Activity rhythms refer to the temporal structuring of behaviors across the 24 h day, enabling animals to optimize foraging opportunities while minimizing exposure to predators [5]. In contrast, activity intensity captures the frequency or magnitude of activity within defined time periods and is influenced by environmental variables such as temperature, soil characteristics, and resource availability [6].

Simultaneous analysis of both components is essential for uncovering mechanisms of temporal niche partitioning, interspecific competition, and species coexistence within ecological communities [7,8,9]. Moreover, these behavioral traits represent long-term evolutionary adaptations to dynamic and fluctuating ecological conditions [10].

Rodents, due to their high sensitivity to environmental changes, exhibit diverse diel activity patterns, including diurnal, nocturnal, crepuscular, and arrhythmic behaviors [11]. In China, a total of 271 rodent species have been recorded (including Rodentia and Lagomorpha), of which 120 species inhabit desert regions, representing 44.28% of the national rodent fauna [12,13]. Among these, *R. opimus* and *M. meridianus* are recognized as dominant or commonly occurring species in the desert ecosystems of northern China [14]. These species play critical ecological roles—contributing to food web stability, soil nutrient cycling, and seed dispersal—while also serving as sensitive indicators of environmental change [15,16,17].

However, when rodent populations exceed ecological thresholds, they can trigger significant ecosystem degradation, including decreased grassland productivity, accelerated soil erosion, and increased public health risks [18,19]. As a result, current rodent management strategies emphasize the dual goals of conserving biodiversity while mitigating the ecological and socioeconomic impacts of rodent outbreaks [20].

This study was conducted in a temperate desert-steppe ecosystem on the northern slopes of the Qilian Mountains—a region characterized by low annual precipitation, sparse vegetation cover, and strong seasonal variability. These environmental conditions are known to strongly shape the activity rhythms of both rodents and their predators.

Obtaining reliable data on animal behavioral dynamics requires robust, field-based methodologies. However, studying activity patterns in small, elusive mammals presents notable logistical and methodological challenges. Traditional methods such as direct behavioral observation [21] and laboratory-based assessments [22] provide useful data but are often constrained by environmental and procedural biases. For example, direct observation is hindered in densely vegetated habitats or during nighttime, where poor visibility and observer presence may alter natural behaviors and compromise data reliability [23]. Additionally, these methods are labor-intensive and time-consuming, limiting their suitability for large-scale or long-term monitoring.

To overcome these limitations, infrared camera traps have become widely used in wildlife research [24]. These devices employ passive infrared sensors to detect motion and heat, enabling continuous, non-invasive monitoring of species presence, behavior, and relative abundance. Camera traps have proven effective in mapping species distributions [25], characterizing activity patterns [26], and estimating population dynamics [27]. Their applications span numerous taxa, including ungulates [28], primates [29], birds [30], and various terrestrial mammals [31,32], underscoring their versatility and reliability.

In recent years, rodent outbreaks and habitat use patterns in this region have been systematically documented as part of the Gansu Grassland Pest Survey Program. These data provided a reliable foundation for identifying representative sites with consistent rodent activity, which formed the basis for our camera trap deployment.

Although prior studies have documented activity rhythms of desert rodents or their predators independently, few have examined multi-species predator–prey interactions within a spatiotemporal framework—particularly in arid steppe ecosystems. Moreover, how divergent activity rhythms between coexisting rodent species influence temporal niche partitioning and their spatiotemporal associations with predators remains inadequately explored [33].

In this study, we simultaneously monitored two ecologically contrasting rodents (*R. opimus*, diurnal; *M. meridianus*, nocturnal) and their primary predators (*O. manul* and *V. vulpes*) using infrared camera traps. Our objectives were to

1. Quantify the daily and seasonal activity rhythms of each species;
2. Assess the temporal and spatial overlap between rodents and predators;
3. Evaluate the potential implications of activity synchrony for natural rodent control in desert-steppe habitats.

By integrating activity rhythm analysis, kernel density estimation, and Pianka’s niche overlap index, this study provides new insights into predator–prey dynamics and offers a potential foundation for biodiversity-friendly rodent management in arid ecosystems.

## 2. Materials and Methods

### 2.1. Study Area

**Figure 1:**
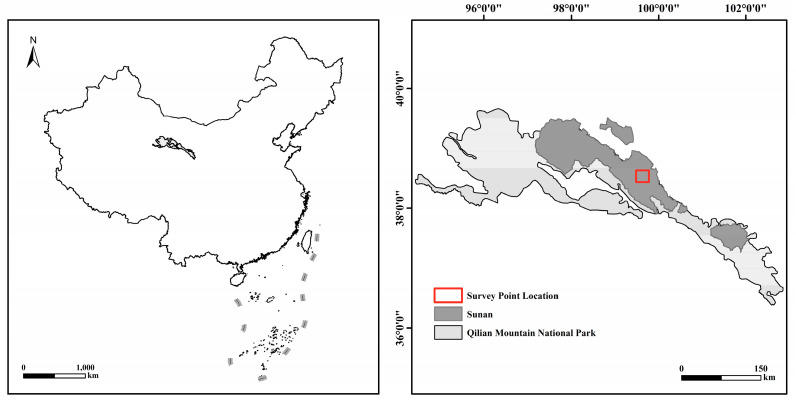
Survey point location.

This study was conducted in a temperate desert-steppe ecosystem in Dahe Township, Sunan County, Gansu Province, China (38°30′–39°15′ N, 99°00′–100°02′ E), at an average elevation of 2230 m (Figure 1). The region experiences an arid climate, with a mean annual temperature of approximately 4 °C and average precipitation of 253 mm, most of which occurs between May and August. The dominant soil type is desert Calcisolsupporting sparse vegetation composed mainly of xerophytic shrubs and semi-shrubs. Representative plant species include *Sympegma regelii*, *Allium polyrhizum*, *Salsola passerina*, *Psathyrostachys juncea*, and *Reaumuria soongorica*. Common resident fauna include *M. meridianus*, *R. opimus*, *V. vulpes*, and *O. manul*.

### 2.2. Infrared Camera Deployment and Maintenance

From December 2020 to January 2022, we deployed 22 infrared-triggered camera traps (Ltl-6210PLUS, Shenzhen Ltl Acorn Electronics Co., Ltd., Shenzhen, China) across representative shrub–grassland habitats in Sunan Yugur Autonomous County. Site selection was informed by long-term rodent distribution data from the Gansu Province Grassland Pest Survey Program, which identified areas with consistently high levels of rodent activity based on fresh burrows, fecal pellets, and feeding traces. The study areas were divided into 100 m × 100 m grids using ArcGIS 10.1 (ESRI Inc., Redlands, CA, USA), and cameras were placed within selected grid cells that showed visible signs of recent rodent presence. The minimum spacing between neighboring cameras was approximately 100 m, corresponding to the estimated home ranges of small desert rodents and minimizing the probability of duplicate detections. Cameras were positioned within 5–10 m of active burrow entrances to maximize the likelihood of capturing both rodent activity and predator foraging behavior. Each camera was mounted at a height of 0.5–1.0 m and angled toward burrow entrances or foraging trails to optimize the detection of both prey and predators.

The cameras were configured to capture three consecutive images per trigger event without delay, providing continuous 24 h monitoring. Maintenance visits were conducted approximately every two months to ensure data integrity and operational consistency through battery replacement and image retrieval.

### 2.3. Species Identification

Captured images were renamed and sorted by species. Species identification was conducted using the Field Guide to the Mammals of China [34] and the Field Guide to the Birds of China [35]. Taxonomic classification followed the Checklist of Mammals in China (2021 Edition) [12] and the Checklist of the Birds of China (3rd Edition) [36]. Species protection statuses were referenced from multiple authoritative sources, including the List of National Key Protected Wild Animals (http://www.forestry.gov.cn, 5 May 2025), the China Bird Red List Assessment [37], the IUCN Red List (http://www.iucnredlist.org, 6 May 2025), and the CITES Appendices (2019, Chinese Edition) (http://www.cites.org.cn, 7 May 2025).

To ensure annotation consistency and minimize observer bias, all images underwent two rounds of independent screening by separate teams. Any discrepancies were resolved through consensus discussions. This dual-stage validation protocol achieved high inter-observer agreement and ensured reliable species-level classification.

### 2.4. Calculation of Activity Intensity

Captured images were screened to identify independent valid photographs (IPs), defined as single detections of a target species at the same site with a minimum 30 min interval. Each 24 h period of camera function was considered one effective trap day.

Two indices were used to quantify activity intensity:

Day Relative Abundance Index (*DRAI*):

$$DRAI=\frac{D_{i}}{N}\times 1000$$

where $D_{i}$ is the number of independent valid photos recorded in hour i (*i* = 0–23), and N is the total number of effective trap days.

Month Relative Abundance Index (*MRAI*):

$$MRAI=\frac{M_{i}}{N_{i}}\times 1000$$

where $M_{i}$ is the number of independent valid photos in month i (i = 1–12), and $N_{i}$ is the number of effective trap days in that month.

To determine activity peaks, we defined peak activity periods as continuous time intervals where the index exceeded 70% of the maximum DRAI or MRAI value. These thresholds were determined empirically and visualized using Origin 2024.

Prior to the statistical summary, data normality was assessed using the one-sample Kolmogorov–Smirnov (K–S) test in IBM SPSS Statistics 26.0.

### 2.5. Temporal Overlap Analysis

Kernel density estimation was used to generate diel activity curves for each species [38]. The degree of temporal overlap was quantified using the overlap coefficient Δ_1_ [39], where Δ_1_ = 0 indicates no overlap and Δ_1_ = 1 indicates complete overlap. The significance of differences in activity distributions was tested using Wald tests for circular data. Analyses and visualizations were conducted using the R packages overlap, activity, and circular. Density curves were visualized with shaded overlap areas to illustrate synchrony patterns.

### 2.6. Spatial Overlap Analysis

Spatial co-occurrence between species was evaluated based on presence–absence data at each of the 22 infrared camera locations. Pianka’s index was used to quantify pairwise spatial niche overlap, calculated as

$$OI=\frac{O_{ij}}{\sqrt{O_{i}\times O_{j}}}$$

where OI is the spatial overlap coefficient; $O_{ij}$ is the number of sites where both species were recorded; and $O_{i},O_{j}$ are the numbers of sites where each species was independently detected.

Data were processed using Microsoft Excel and Python 3.11 (Pandas, Numpy, Matplotlib). To enhance visual clarity, an AI-based image generation tool (ChatGPT-4) was used to assist in the illustration (Figure 6). The generated image was reviewed and edited by the authors to ensure scientific accuracy.

## 3. Results

### 3.1. Species Detection and Data Overview

From December 2020 to January 2022, a total of 8052 effective trap-days were recorded using 22 infrared cameras across three rodent-infested sites. The cameras captured 553,814 images, among which 28,881 were successfully identified to the species level, yielding a recognition rate of 5.2%.

**Figure 2:**
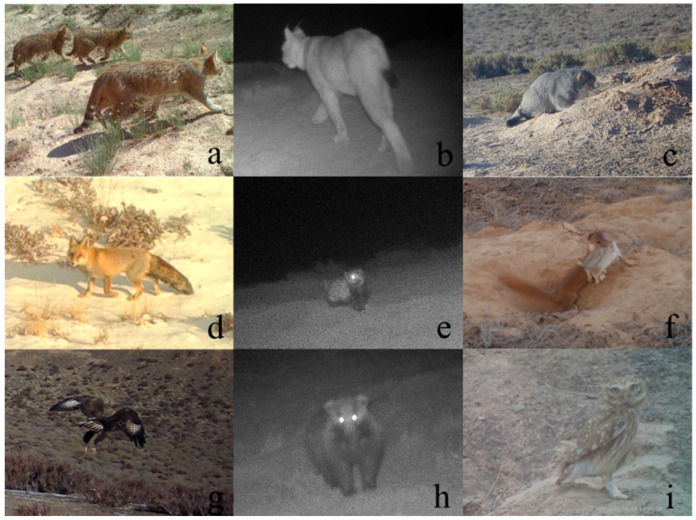
Representative images of predators captured by infrared cameras. Note: (**a**) *Felis bieti*, (**b**) *Lynx lynx*, (**c**) *Otocolobus manul*, (**d**) *Vulpes vulpes*, (**e**) *Vormela peregusna*, (**f**) *Mustela nivalis*, (**g**) *Aquila nipalensis*, (**h**) *Meles leucurus*, (**i**) *Athene noctua*.

The dataset included two focal rodent species—*R. opimus* and *M. meridianus*—alongside nine predator species spanning five families and two taxonomic orders. Among these predators, seven were mammals and two were birds. The most frequently detected predator was *O. manul*, followed by *V. vulpes* and the Least weasel (*Mustela nivalis*), Chinese desert cat (*Felis bieti*), Eurasian lynx (*Lynx lynx*), Marbled polecat (*Vormela peregusna*), Steppe eagle (*Aquila nipalensis*), Asian Badger (*Meles leucurus*), and Little owl (*Athene noctua*) (Figure 2).

To examine predator–prey spatiotemporal dynamics in greater detail, we focused on the four most frequently detected species: *R. opimus*, *M. meridianus*, *O. manul*, and *V. vulpes*. A total of 26,212 valid images and 3403 independent detection events were recorded for these species, including 25,215 images and 3143 events for *R. opimus*, 754 images and 197 events for *M. meridianus*, 159 images and 34 events for *O. manul*, and 84 images and 29 events for *V. vulpes* (Table 1).

### 3.2. Daily and Monthly Activity Rhythms of R. opimus and M. meridianus

The daily and monthly activity patterns of the two focal rodent species were analyzed using data from March 2021 to February 2022. Two indices were applied: the Daily Relative Abundance Index (DRAI) and the Monthly Relative Abundance Index (MRAI).

**Figure 3:**
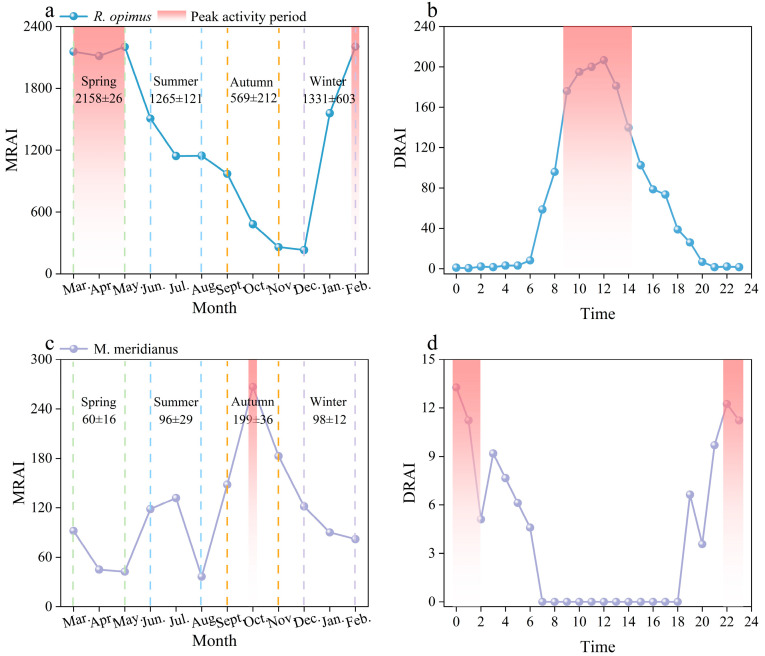
Daily and monthly activity intensity of *R. opimus* and *M. meridianus*. Note: Red shaded areas represent peak activity periods, defined as intervals exceeding 70% of the maximum DRAI or MRAI value. Time is displayed on a 24 h scale. Seasons follow Northern Hemisphere climatic definitions: Spring (March–May), Summer (June–August), Autumn (September–November), and Winter (December–February). (**a**) Monthly variation in activity intensity of *R. opimus* based on MRAI. (**b**) Daily activity intensity of *R. opimus* across seasons based on DRAI. (**c**) Monthly variation in activity intensity of *M. meridianus* based on MRAI. (**d**) Daily activity intensity of *M. meridianus* across seasons based on DRAI.

As illustrated in Figure 3a,b, *R. opimus* displayed a unimodal diurnal activity rhythm, with activity beginning at approximately 06:00, peaking between 09:00 and 14:00, and declining sharply thereafter. Very limited activity was recorded after 18:00. On a seasonal scale, *R. opimus* showed sustained high activity from February through May, followed by a decline in summer and autumn.

In contrast, *M. meridianus* exhibited a clearly bimodal nocturnal pattern, with activity concentrated during nighttime hours, particularly between 22:00 and 02:00 (Figure 3d). Almost no activity was recorded from 08:00 to 18:00, affirming its strictly nocturnal behavior. Its monthly activity exhibited greater variability, with a distinct peak in October (Figure 3c), suggesting a more episodic and seasonally constrained activity profile.

### 3.3. Temporal Overlap Between Rodents and Their Predators

The temporal activity overlap between rodents and their predators was evaluated using kernel density curves and the overlap coefficient (Δ), at both daily and monthly scales.

**Figure 4:**
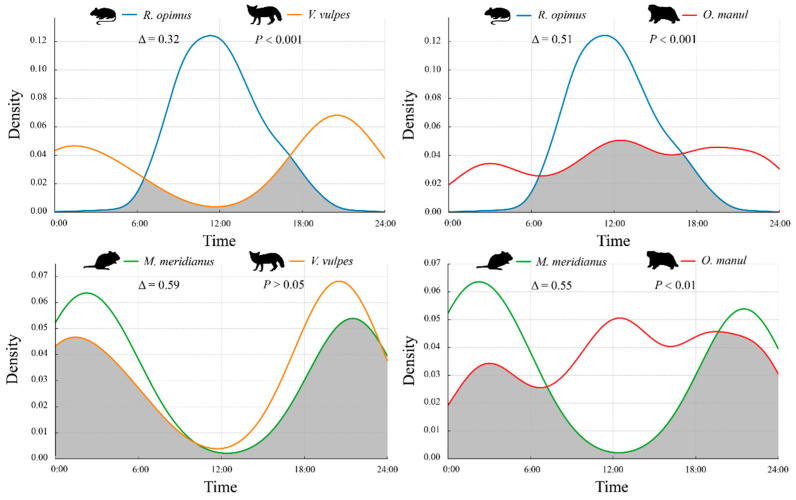
Daily activity rhythm overlap between rodents and their predators. Note: Shaded areas indicate overlapping time periods. Δ denotes the overlap coefficient; *p*-values reflect the significance of activity distribution differences.

As shown in Figure 4, *R. opimus* was active during daylight hours (peak: 09:00–14:00), while *V. vulpes* showed a crepuscular–nocturnal pattern, with moderate peaks at dawn and dusk. The resulting daily overlap was low (Δ = 0.32, *p* < 0.001), suggesting temporal avoidance. In contrast, *O. manul* displayed a broad bimodal rhythm with sustained activity from late morning to early evening, resulting in moderate daily overlap with *R. opimus* (Δ = 0.51, *p* < 0.001).

*M. meridianus* showed a strict nocturnal pattern with two pronounced peaks—pre-midnight and pre-dawn—substantially overlapping with *V. vulpes* (Δ = 0.59, *p* > 0.05) and *O. manul* (Δ = 0.55, *p* < 0.01), driven by their synchronized nighttime activity.

**Figure 5:**
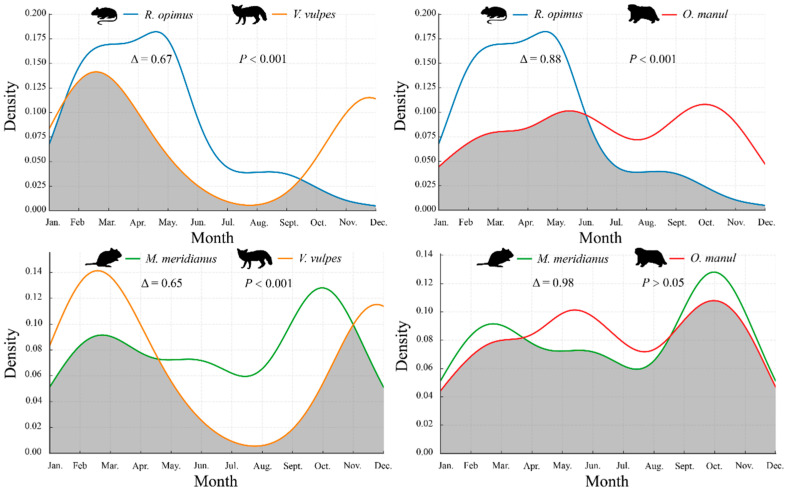
Monthly activity rhythm overlap between rodents and their predators.

At the monthly scale (Figure 5), *R. opimus* was most active from February to May, partially overlapping with *V. vulpes* (Δ = 0.67, *p* < 0.001), which had seasonal peaks in late winter and autumn. A higher degree of seasonal synchrony was observed between *R. opimus* and *O. manul* (Δ = 0.88, *p* < 0.001), reflecting the predator’s extended activity across spring and summer.

*M. meridianus* exhibited bimodal seasonal peaks in spring and autumn, overlapping with *V. vulpes* (Δ = 0.65, *p* < 0.001) and showing near-complete synchrony with *O. manul* (Δ = 0.98, *p* > 0.05).

### 3.4. Spatial Distribution and Overlap Between Rodents and Their Predators

**Figure 6:**
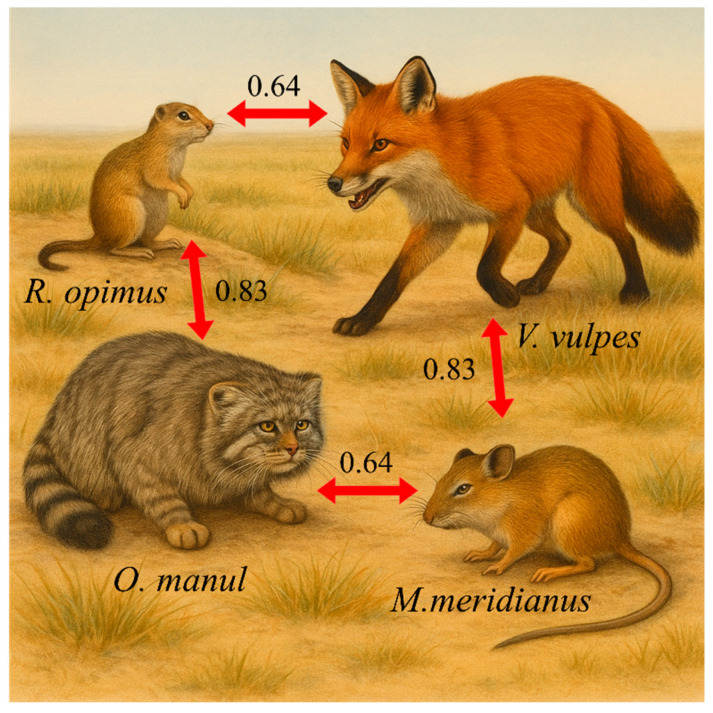
Spatial overlap between rodents and predators. Note: Values represent pairwise spatial overlap indices (OI), based on the proportion of shared detection sites recorded by infrared cameras.

The spatial overlap coefficients (OI values), derived from shared camera detection sites, revealed consistently high spatial associations between focal rodents and their predators (Figure 6). High spatial overlap was observed between *R. opimus* and *O. manul* (OI = 0.83) and between *M. meridianus* and *V. vulpes* (OI = 0.83). Moderate yet equal values were recorded for *R. opimus*–*V. vulpes* and *M. meridianus*–*O. manul* (both OI = 0.64).

## 4. Discussion

### 4.1. Differences in Activity Rhythms of Rodents

Temporal niche differentiation is a key mechanism promoting coexistence among ecologically similar species. In this study, *R. opimus* and *M. meridianus* exhibited distinct diel activity rhythms—diurnal and nocturnal, respectively—which minimize direct competition and reflect divergent adaptive strategies in a resource-limited environment [40,41].

*R. opimus* showed consistent daytime activity, with a unimodal peak from 09:00 to 14:00, and sustained seasonal activity from February to May. This contrasts with previous findings of autumnal activity peaks [19], likely due to regional climatic variation or habitat use shifts [42].

*M. meridianus* maintained a stable nocturnal pattern, peaking between 22:00 and 02:00, with seasonal activity concentrated in October. This timing may be linked to increased energetic demands for reproduction or fat accumulation in preparation for winter [43]. The adjustment of activity timing across seasons suggests that the photoperiod plays a regulatory role in its behavioral rhythms, consistent with adaptations seen in other nocturnal desert rodents.

Similar diel segregation has been observed among sympatric rodent species inhabiting other arid environments. For instance, in the Alxa Desert of northern China, *M meridianus* and *Dipus sagitta*—two ecologically similar nocturnal rodents—displayed fine-scale temporal partitioning within the night, with different peak activity windows, which likely facilitates their stable coexistence despite overlapping diets and habitat use [44]. Experimental evidence from small mammals in North American deserts further supports the role of diel rhythm differentiation in resource-limited systems. *Peromyscus* species, for example, show photoperiod-entrained activity rhythms that anticipate daily environmental changes, thereby aligning metabolic and behavioral strategies with resource availability [45].

These studies collectively suggest that temporal niche differentiation—whether expressed through distinct day–night patterns or intra-night partitioning—represents a widespread mechanism by which desert-dwelling small mammals reduce competition, mitigate environmental stress, and coexist in resource-scarce habitats.

### 4.2. Spatiotemporal Interactions with Predators

Predation risk is shaped by both spatial proximity and temporal synchrony between predators and prey. Our results revealed differing interaction potentials among the four focal species. *M. meridianus* exhibited high spatiotemporal overlap with both *V. vulpes* (Δ_day = 0.59; Δ_month = 0.65; OI = 0.83) and *O. manul* (Δ_day = 0.55; Δ_month = 0.98; OI = 0.64), suggesting a high probability of encounter.

By contrast, *R. opimus* displayed a lower daily overlap with *V. vulpes* (Δ = 0.32), which may reduce predation risk via diel avoidance. However, its overlap with *O. manul* was higher both daily (Δ = 0.51) and seasonally (Δ = 0.88), likely due to *O. manul*’s partially diurnal activity and strong spatial co-occurrence (OI = 0.83).

The activity pattern of *O. manul* in this study diverges from its reported nocturnality in other habitats [16,46], suggesting behavioral flexibility aligned with prey availability—particularly the diurnal behavior of *R. opimus*. In contrast, *V. vulpes* retained its typical crepuscular–nocturnal rhythm, with increased seasonal activity likely linked to altitudinal migration during colder months [6,47].

Similar spatiotemporal predator–prey dynamics have been observed in other arid regions. In the Negev Desert, gerbils reduce predation risk by shifting activity away from peak predator hours and brighter moon phases, demonstrating fine-scale temporal avoidance [48]. Likewise, in Australian desert systems, nocturnal rodents such as *Notomys alexis* exhibit flexible spatial behavior under predator pressure, balancing escape potential with foraging needs [49].

These patterns echo our findings: *R. opimus* may mitigate risk from *V. vulpes* through diel segregation, while *O. manul* appears to adjust its temporal activity to align with prey availability. Collectively, these cases highlight behavioral plasticity as a critical survival strategy in desert predator–prey systems.

### 4.3. Ecological Implications and Management Prospects

The observed high spatial overlap between predators and rodent prey (OI = 0.64–0.83) underscores the potential for enhancing natural predation in rodent population regulation. Notably, strong spatial and temporal alignment between *O. manul* and *R. opimus*, and between *V. vulpes* and *M. meridianus*, suggests that predator behavioral responses are tightly coupled with prey activity patterns.

These findings support the development of predator-based strategies for regulating rodent populations, especially in arid and semi-arid grasslands where conventional chemical control may be ecologically disruptive. The strong spatial and temporal associations we observed—particularly between *R opimus* and *O manul*, and between *M meridianus* and *V vulpes*—suggest that native carnivores may exert a consistent top-down pressure on dominant rodent species.

By aligning their activity rhythms and foraging ranges with those of their prey, these predators can maximize encounter rates, potentially suppressing rodent outbreaks through direct predation or altered rodent behavior (e.g., reduced foraging, increased vigilance). This behavioral linkage highlights the potential for enhancing natural predation as a biodiversity-friendly rodent management tool.

However, spatiotemporal overlap alone cannot confirm predation. Future research should integrate dietary assessments (e.g., scat analysis, stable isotopes, DNA metabarcoding) to verify actual trophic relationships [32]. Long-term monitoring is also needed to evaluate how habitat heterogeneity and environmental fluctuations influence predator–prey dynamics at broader spatial scales.

While our infrared camera trap approach offered valuable insights into species interactions, we acknowledge that certain methodological biases may have influenced our findings. First, camera traps were primarily placed near active burrows or foraging trails, which may have inflated detection probabilities for rodents and predators frequenting these areas, while underrepresenting less active or peripheral zones.

Second, spatial clustering within high-activity microhabitats may have introduced sampling bias, potentially exaggerating overlap indices. Additionally, species with habitual movement patterns along predictable routes may be overrepresented due to repeated detections at a single location.

Future studies should consider randomized or stratified camera placement across multiple habitat types, incorporate detection probability models, and integrate complementary methods such as line transects or direct observation to mitigate these limitations.

## 5. Conclusions

This study revealed distinct diel and seasonal activity patterns in two sympatric desert rodents—*R. opimus* (diurnal) and *M. meridianus* (nocturnal)—demonstrating clear temporal niche differentiation that facilitates coexistence. Despite these differences, both species exhibited substantial spatiotemporal overlap with their key predators, *O. manul* and *V. vulpes*. Notably, *O. manul* displayed flexible activity rhythms that closely aligned with the behavior of *R. opimus*, suggesting adaptive predation strategies. These findings highlight the ecological importance of behavioral synchrony in predator–prey systems and suggest that enhancing native predator populations may offer a viable, biodiversity-friendly approach to rodent management in arid grasslands. Future efforts should integrate behavioral monitoring with dietary evidence to validate predation outcomes and inform ecologically grounded pest control strategies.

## Figures and Tables

**Table 1:** Valid images and independent detection events for focal species.

Species	Total_Images	Independent_Events
*R. opimus*	25,215	3143
*M. meridianus*	754	197
*V. vulpes*	84	29
*O. manul*	159	34

## Data Availability

The datasets used and/or analyzed during the current study are available from the corresponding author on reasonable request.

## Abbreviations

The following abbreviations are used in this manuscript:

IP	Independent Photographic Event
DRAI	Daily Relative Activity Index
MRAI	Monthly Relative Activity Index
OI	Spatial Overlap Index
Δ	Temporal Overlap Coefficient

## References

[1] Allada R., White N.E., So W.V., Hall J.C., Rosbash M. (1998). A mutant Drosophila homolog of mammalian Clock disrupts circadian rhythms and transcription of period and timeless. Cell.

[2] Nie Y., Speakman J.R., Wu Q., Zhang C., Hu Y., Xia M., Yan L., Hambly C., Wang L., Wei W. (2015). Exceptionally low daily energy expenditure in the bamboo-eating giant panda. Science.

[3] Zhou S., Zhang J., Hull V., Huang J., Liu D., Zhou J., Sun M., Zhang H. (2019). Comparative activity patterns of wild giant pandas and livestock. Acta Ecol. Sin..

[4] Probst R., Probst R. (2023). High frequency of Apodemus mice boosts inverse activity pattern of bank voles, *Clethrionomys glareolus*, through non-aggressive intraguild competition. Animals.

[5] Kronfeld-Schor N., Bloch G., Schwartz W.J. (2013). Animal clocks: When science meets nature. Proc. R. Soc. B Biol. Sci..

[6] Kronfeld-Schor N., Dayan T. (2003). Partitioning of time as an ecological resource. Annu. Rev. Ecol. Evol. Syst..

[7] Dominoni D.M., Åkesson S., Klaassen R., Spoelstra K., Bulla M. (2017). Methods in field chronobiology. Philos. Trans. R. Soc. B.

[8] Oliveira-Santos L.G.R., Zucco C.A., Agostinelli C. (2013). Using conditional circular kernel density functions to test hypotheses on animal circadian activity. Anim. Behav..

[9] Su X., Li X., Sun H., Song Z. (2024). Analysis of activity rhythm and behavior pattern for plateau pika in degraded alpine meadow. Discov. Appl. Sci..

[10] Zhao L.J., Liu M.Z., Luo C.P. (2020). Daily activity rhythm of *Ithaginis cruentus* in the Wanglang National Nature Reserve. Sichuan J. Zool..

[11] Daan S., Aschoff J., Aschoff J., Daan S., Groos G.A. (1982). Circadian Contributions to Survival. Vertebrate Circadian Systems: Structure and Physiology.

[12] Wei F., Yang Q., Wu Y., Jiang X., Liu S., Li B., Yang G., Li M., Zhou J., Li S. (2021). Catalogue of Mammals in China (2021). Acta Theriol. Sin..

[13] Zhou L.-Z., Ma Y. (2002). Distribution Patterns of Rodent Diversity in Arid Regions of West China. Biodivers. Sci..

[14] Zhou L., Ma Y., Li D.L. (2001). Spatial Distribution Patterns of Chinese Gerbils (Gerbillinae) in Relation to Environmental Factors. Acta Zool. Sin..

[15] Flowerdew J.R., Shore R.F., Poulton S.M.C., Sparks T.H. (2004). Live Trapping to Monitor Small Mammals in Britain. Mammal Rev..

[16] Davidson A.D., Detling J.K., Brown J.H. (2012). Ecological Roles and Conservation Challenges of Social, Burrowing, Herbivorous Mammals in the World’s Grasslands. Front. Ecol. Environ..

[17] Sunyer P., Muñoz A., Bonal R., Espelta J.M. (2013). The Ecology of Seed Dispersal by Small Rodents: A Role for Predator and Conspecific Scents. Funct. Ecol..

[18] Kausrud K.L., Viljugrein H., Frigessi A., Begon M., Davis S., Leirs H., Dubyanskiy V., Stenseth N.C. (2007). Climatically Driven Synchrony of Gerbil Populations Allows Large-Scale Plague Outbreaks. Proc. R. Soc. B Biol. Sci..

[19] Wen X., Cheng X., Dong Y., Wang Q., Lin X. (2020). Analysis of the Activity Rhythms of the Great Gerbil (*Rhombomys opimus*) and Its Predators Based on Infrared Camera Technology. Glob. Ecol. Conserv..

[20] Hua L.M., Chai S.Q. (2022). Rodent Pest Control on Grasslands in China: Current State, Problems and Prospects. J. Plant Prot..

[21] Kuhn K.M., Vander Wall S.B. (2008). Linking Summer Foraging to Winter Survival in Yellow Pine Chipmunks (*Tamias amoenus*). Oecologia.

[22] Smale L., Lee T., Nuñez A.A. (2003). Mammalian Diurnality: Some Facts and Gaps. J. Biol. Rhythm..

[23] Rowcliffe J.M., Carbone C. (2008). Surveys Using Camera Traps: Are We Looking to a Brighter Future?. Anim. Conserv..

[24] Cappelle N., Després-Einspenner M., Howe E.J., Boesch C., Kühl H.S. (2019). Validating Camera Trap Distance Sampling for Chimpanzees. Am. J. Primatol..

[25] Ferreguetti Á.C., Tomás W.M., Bergallo H.G. (2015). Density, Occupancy, and Activity Pattern of Two Sympatric Deer (*Mazama* spp.) in the Atlantic Forest, Brazil. J. Mammal..

[26] Gerber B.D., Karpanty S.M., Randrianantenaina J. (2012). Activity Patterns of Carnivores in the Rain Forests of Madagascar: Implications for Species Coexistence. J. Mammal..

[27] Srbek-Araujo A.C., Silveira L.F., Chiarello A.G. (2012). The Red-Billed Curassow (*Crax blumenbachii*): Social Organization, and Daily Activity Patterns. Wilson J. Ornithol..

[28] Oliveira-Santos L.G.R., Tortato M.A., Graipel M.E. (2008). Activity Pattern of Atlantic Forest Small Arboreal Mammals as Revealed by Camera Traps. J. Trop. Ecol..

[29] Galetti M., Camargo H., Siqueira T., Keuroghlian A., Donatti C.I., Jorge M.L.S.P., Pedrosa F., Kanda C.Z., Ribeiro M.C. (2015). Diet Overlap and Foraging Activity between Feral Pigs and Native Peccaries in the Pantanal. PLoS ONE.

[30] Tang X., Tang S., Li X., Menghe D., Bao W., Xiang C., Gao F., Bao W. (2019). A Study of Population Size and Activity Patterns and Their Relationship to the Prey Species of the Eurasian Lynx Using a Camera Trapping Approach. Animals.

[31] Smith A.T., Yan X. (2013). Distribution and Identification of Chinese Mammals. Chin. Mammal. J..

[32] MacKinnon J.R., Phillipps K. (2000). Illustrated Guide to the Birds of China. Chin. Avian J..

[33] Zheng G. (2005). Checklist on the Classification and Distribution of the Birds of China. Avian Taxon. Bull. China.

[34] Zhang Y.-Y., Zhang Z.-W., Dong L., Ding P., Ding C.-Q., Ma Z.-J., Zheng G.-M. (2016). Assessment of Red List of Birds in China. Biodivers. Sci..

[35] Chen L.J., Shu Z.F., Xiao Z.S. (2019). Application of Camera-Trapping Data to Study Daily Activity Patterns of Galliformes in Guangdong Chebaling National Nature Reserve. Sci. Silvae Sin..

[36] Azevedo F.C., Lemos F.G., Freitas-Junior M.C., Rocha D.G., Azevedo F.C.C. (2018). Puma Activity Patterns and Temporal Overlap with Prey in a Human-modified Landscape at Southeastern Brazil. J. Zool..

[37] Guo Q., Zuo Y., Lin W., Xiao X., Zhao X., Li Y., Ying Z., Zhou C., Xie X. (2022). Niche and Interspecific Association of Juvenile *Tachypleus tridentatus* in the Beibu Gulf. Acta Oceanol. Sin..

[38] Namgail T., Fox J.L., Bhatnagar Y.V. (2004). Habitat Segregation between Sympatric Tibetan Argali (*Ovis ammon hodgsoni*) and Blue Sheep (*Pseudois nayaur*) in the Indian Trans-Himalaya. J. Zool..

[39] Qiao H.H., Liu W., Yang W.K., Xü W.X. (2011). Behavioral Ecology of *Rhombomys opimus*: A Review. Chin. J. Ecol..

[40] Lin J., Zhang X., Wang C. (2006). Demography and Reproduction of *Meriones meridianus* in Inner Mongolia. Endem. Dis. Bull..

[41] Zhao D., Yang C.M., He M.X., Chen L.X., He X.C., Ran J.H. (2019). Habitat Suitability Assessment and Daily Activity Patterns of *Otocolobus manul* in the Gongga Mountain National Nature Reserve. Sichuan J. Zool..

[42] Murdoch J.D., Munkhzul T., Reading R.P. (2006). Pallas’s Cat Ecology and Conservation in the Semi-Desert Steppes of Mongolia. Cat News.

[43] Shi X.H., Hu Q.U., Feng X., Jin S.H., Cheng Y., Zhang J., Yao M.A., Li S.L. (2021). Spatiotemporal Relationships between Snow Leopard (*Panthera uncia*) and Red Fox (*Vulpes vulpes*) in Qionglai Mountains, Sichuan Province. Acta Theriol. Sin..

[44] Li X., Yuan S., Li L., Wang J., Ma J., Ji Y., Lu X. (2023). Influence of Grazing on the Activity Pattern and Temporal Niche of Two Dominant Rodent Species in Alxa Desert. Front. Ecol. Evol..

[45] Colella J.P., Blumstein D.M., MacManes M.D. (2021). Disentangling Environmental Drivers of Circadian Metabolism in Desert-Adapted Mice. J. Exp. Biol..

[46] Zhang H., Li C., Dou H., Liu S., Wang M. (2012). Red Fox Habitat Selection and Landscape Feature Analysis in the Dalai Lake Natural Reserve in Inner Mongolia. Acta Ecol. Sin..

[47] Burt S.A., Lipman S.A. (2021). What Do They Know? Comparing Public Knowledge and Opinions about Rodent Management to the Expectations of Pest Controllers. Animals.

[48] Kotler B.P., Ayal Y., Subach A. (1994). Effects of Predatory Risk and Resource Renewal on the Timing of Foraging Activity in a Gerbil Community. Oecologia.

[49] Spencer E.E., Crowther M.S., Dickman C.R. (2014). Risky Business: Do Native Rodents Use Habitat and Odor Cues to Manage Predation Risk in Australian Deserts?. PLoS ONE.

